# Mental health without belonging: the crisis of relational safety in digital-native emerging adults

**DOI:** 10.3389/fpsyt.2025.1738103

**Published:** 2026-01-21

**Authors:** Ginto Chirayath, Anithamol Babu

**Affiliations:** 1 Bishop Appaswamy College of Arts and Science, Coimbatore, India; 2 Marian College Kuttikkanam Autonomous, Kuttikkanam, India

**Keywords:** AI ethics, belonging, digital mental health, ecological psychiatry, emerging adulthood, policy intervention, relational safety, youth well-being

Emerging adulthood, spanning the ages of eighteen to thirty years, has become a profoundly fragile developmental stage. Once idealised as a time of opportunity and self-definition, it now unfolds within a global environment of instability—marked by economic precarity, environmental distress, and constant digital immersion. Epidemiological evidence indicates that nearly two-thirds of lifetime mental disorders emerge before age twenty-five, with the burden of anxiety and depression intensifying in the post-pandemic era (1, 2). Yet these figures obscure a deeper, structural fracture: the erosion of belonging. In today’s hyperconnected world, communication is abundant, but relational safety—the confidence that one’s vulnerabilities will be received with empathy and without judgment—has become uncertain. This crisis is not simply emotional but infrastructural, shaping how individuals relate to self and others. Psychiatry must therefore reimagine belonging not as sentiment but as a public-health determinant and a foundation for prevention (3). As an opinion article, this article advances a conceptual and policy-oriented framework rather than reporting original empirical findings; its primary aim is to synthesise existing evidence, identify structural gaps, and propose testable directions for future research and intervention. The concept of relational safety advanced in this article is an author-derived, integrative construct rather than a formally standardised diagnostic or policy definition. It extends adjacent concepts such as social support, therapeutic alliance, and psychological safety by shifting analytical focus from context-bound relationships to the ambient relational conditions that enable vulnerability, trust, and help-seeking across everyday digital, institutional, and community environments. Grounded in established empirical and theoretical literatures—including attachment theory, psychological safety research, therapeutic alliance scholarship, and social-ecological psychiatry—this construct integrates relational processes across levels of analysis and reflects informed scholarly judgement anchored in adjacent evidence bases rather than an assumed or universal consensus.

The digital ecosystem has increasingly shaped the psychological architecture of emerging adulthood, influencing how belonging, trust, and identity are negotiated rather than determining them in a uniform or deterministic manner (4). While technology facilitates contact, it often intensifies loneliness, mistrust, and emotional comparison. Meta-analytic findings show that nearly half of young adults experience cyber-dating abuse as victims or perpetrators (5). Technology-facilitated gender-based violence is associated with anxiety, depressive symptoms, and social withdrawal (6). Cyberbullying and cyberstalking predict self-harm and emotional dysregulation (7–9). Social media, optimised for attention rather than authenticity, transforms belonging into a metric of visibility (10). In India and similar Global South contexts, digital risk is compounded by class inequality, patriarchal family systems, and limited mental-health infrastructure (11). The cumulative effect is a landscape where connection is transactional, and belonging is a fragile, unevenly distributed psychological commodity.

The framework of relational safety provides both interpretive coherence and operational value, while also inviting critical reflection on whether its four proposed dimensions fully capture culturally variant forms of belonging in collectivist, interdependent, or non-Western social contexts. Here, relational safety is advanced as an integrative heuristic construct, synthesising insights from attachment theory, psychological safety, and social-ecological psychiatry, rather than as a fully validated standalone metric. Rooted in attachment theory and psychological-safety models, relational safety can be conceptualised through four measurable dimensions—trust, predictability, reciprocity, and recognition—that together sustain emotional regulation and resilience (12). Trust reduces anticipatory threat, predictability provides stability, reciprocity ensures balance, and recognition validates worth. Digital ecosystems disrupt each of these dimensions through anonymity, technoference, and algorithmic unpredictability (13–15). Offline relational density—sustained embodied contact—acts as a protective buffer (16). The task ahead is to operationalise relational safety empirically and to develop digital, institutional, and community systems that can measure and strengthen it. Specifically, future empirical work should map these dimensions onto established measures such as attachment security scales, perceived social support indices, therapeutic alliance instruments, and social trust metrics, thereby rendering the construct testable and comparable across settings.

Reframing mental health as an ecological phenomenon broadens psychiatry’s scope beyond clinical symptomatology to structural causation, while also raising critical questions about how mechanisms of change can be specified and operationalised across interacting ecological levels. Drawing on Bronfenbrenner’s (17) ecological model, the crisis of belonging can be located at the intersection of personal, institutional, and structural domains. At the individual level, algorithmic comparison and fragmented attention undermine identity and intimacy. At the institutional level, education and employment systems reward productivity over empathy. At the structural level, inequality, climate anxiety, and digital capitalism intensify precarity. The mental health of emerging adults, therefore, represents not only an individual vulnerability but an ecological failure—one requiring systems-level intervention.

To restore belonging, a three-phase intervention model is proposed that aligns with feasibility, acceptability, efficacy, safety, and ethical compliance benchmarks, with all effectiveness claims remaining hypothetical and contingent on future empirical testing through pilot studies and controlled trials. This model is intentionally presented as a feasibility-informed, policy-relevant blueprint rather than an implementation-ready programme, recognising the need for phased piloting, contextual adaptation, and implementation-science evaluation before scale-up. Phase I: Capacity Building in Education involves embedding emotional literacy, digital hygiene, and relational-safety training into school and university curricula. Teachers, counsellors, and parents would undergo modular training on trauma-informed communication and digital well-being. Feasibility is high, as these initiatives can be integrated within existing frameworks like India’s National Education Policy (2020). Acceptability is supported by pilot evidence that youth co-designed psychosocial programmes foster greater engagement (18). Phase II: Community Infrastructure for Belonging focuses on scalable, low-cost relational support networks. Local mental-health clubs, youth collectives, and peer-support systems can operate through existing community structures (e.g., Kudumbashree, ASHA, and panchayat networks). Clinicians and social workers would act as supervisors, ensuring fidelity and safety. Evaluation of efficacy would rely on pre–post measures of stress, help-seeking, and relational trust using validated tools (PHQ-9, GAD-7, UCLA Loneliness Scale). Phase III: Hybrid Digital Systems integrates AI-enabled early detection with human follow-up. Relational-safety dashboards, built using anonymised data streams from voluntary users, could detect social withdrawal, cyberbullying exposure, or linguistic distress cues and automatically route support requests to trained human responders via Tele-MANAS or KIRAN helplines (19).

To establish feasibility, pilot deployment could begin in five Indian states—Kerala, Karnataka, Tamil Nadu, Maharashtra, and Delhi—chosen for their digital readiness and mental-health infrastructure. Data would be collected through encrypted mobile interfaces compliant with India’s Digital Personal Data Protection Act (2023). Algorithms for detection would employ Natural Language Processing (NLP) models trained on multilingual, anonymised text datasets representing regional linguistic diversity. Validation metrics could include precision, recall, and F-scores for identifying high-risk messages. For acceptability, participatory design workshops involving youth, clinicians, and community leaders would guide user-interface and consent features. Youth participation is positioned as integral to the governance and knowledge-production processes of the intervention rather than as a supplementary design input, with power asymmetries between emerging adults, clinicians, and institutions addressed through structured co-decision arrangements, clearly defined roles, and transparent accountability mechanisms. Emerging adults are positioned as active contributors across all phases of the intervention lifecycle, including problem formulation, framework development, data interpretation, evaluation, and ethical governance. Such sustained engagement strengthens both the legitimacy and the epistemic validity of the framework, ensuring that constructs such as relational safety remain grounded in lived experience rather than abstracted expert assumptions. Such participatory approaches are essential to centre the lived experiences and agency of emerging adults, ensuring that systems designed to enhance belonging are co-produced with, rather than imposed upon, the populations they intend to serve. Feedback loops through qualitative interviews and in-app surveys would measure perceived trust and cultural appropriateness. Efficacy would be evaluated through randomised or quasi-experimental designs measuring improvements in early-risk identification and reductions in reported distress over six- and twelve-month periods. Safety mechanisms would include manual human verification before escalation, real-time audit trails for all automated alerts, and built-in “opt-out” options to preserve user autonomy. Independent ethics boards would conduct quarterly reviews to monitor unintended harms, such as false positives or emotional fatigue.

Ethical compliance must be built into system architecture, not appended as afterthought, while acknowledging that continuous ethical auditing can be resource-intensive, particularly within low-resource public mental health systems. Informed consent should be iterative, allowing users to modify or withdraw participation easily. All datasets must undergo multilayered anonymisation—removing identifiable markers before algorithmic processing—and differential-privacy techniques can prevent re-identification. A dedicated Ethical Oversight Council for Digital Mental Health could coordinate with existing agencies under the Digital Personal Data Protection Act and the Ministry of Health. Its mandate would include periodic audits, community representation, and public disclosure of compliance reports. The council would establish accountability through transparent metrics—frequency of ethical breaches, resolution timelines, and user satisfaction indices.

Bias mitigation strategies are essential to ensure that digital interventions do not reproduce social inequities, while recognising that fairness metrics themselves are contested, culturally contingent, and normatively laden, thereby placing epistemic limits on purely technical approaches to bias mitigation. Algorithmic fairness audits should measure representational balance across gender, language, caste, and regional categories. Model training should employ counterfactual fairness techniques, ensuring predictions remain consistent when sensitive attributes are altered. Continuous bias detection pipelines could flag disparities in model outcomes using metrics such as demographic parity and equal opportunity difference. Cultural-linguistic adaptation can be achieved through community data partnerships that allow AI systems to learn from regionally grounded idioms of distress. Independent technical and social-science teams should co-lead audits to ensure epistemic inclusivity.

Sustainability depends on a robust funding and policy framework. A blended financing model could be adopted: government funding for educational and public-health integration; CSR partnerships in technology and healthcare sectors for digital platform development; and international collaboration (WHO, UNICEF, Wellcome Trust) for research and capacity-building grants. Early-stage implementation can be financed through reallocation of existing Tele-MANAS budgets, supplemented by competitive research grants under India’s Department of Science and Technology or ICMR. Evaluation should follow a stepwise adaptive model: pilot > evaluation > refinement > national scale-up. Cost-effectiveness analyses using quality-adjusted life years (QALY) and cost-per-risk-reduction indices can guide resource allocation. Integration with WHO’s Comprehensive Mental Health Action Plan (2023–2030) would further align funding streams with global accountability structures.

Operationalising such a multi-layered model will face practical and ethical challenges. Cultural stigma continues to constrain help-seeking; long-term social-marketing campaigns that reframe vulnerability as collective strength are crucial. Unequal digital access requires low-bandwidth, multilingual platforms with offline extensions via SMS or community radio. Institutional inertia within education and healthcare systems can be overcome through performance-linked incentives for schools and districts demonstrating mental-health outcomes. Ethical risks related to surveillance or overreach must be pre-empted through participatory governance—embedding user councils and citizen juries in oversight processes. The success of these interventions will rely on cross-sectoral collaboration and on ensuring that technological sophistication does not eclipse human empathy.

Future research must refine and empirically validate relational safety as both concept and practice, with early empirical efforts prioritising qualitative and mixed-methods designs—such as ethnographic, phenomenological, and participatory studies—to establish construct validity and capture how trust, recognition, and reciprocity are negotiated in digitally mediated contexts. Subsequent longitudinal and quasi-experimental designs can then examine change over time and inform the development of scalable intervention trials. Developing a standardised Relational Safety Scale will allow quantitative monitoring of trust, predictability, reciprocity, and recognition. Longitudinal and mixed-methods studies can track how these indicators evolve across digital exposure levels, socioeconomic status, and cultural settings. Comparative research across low- and middle-income countries can identify context-specific protective factors such as intergenerational caregiving or collective rituals that preserve belonging despite technological disruption. From a policy-oriented perspective, relational-safety indicators could be analytically aligned with existing WHO mental-health monitoring domains—such as social support, community connectedness, psychosocial well-being, and rights-based care—as a way of rendering belonging measurable rather than purely philosophical (1, 20). This proposal is advanced here as an informed conceptual extension, not as an established or WHO-endorsed indicator set, and is intended to stimulate future empirical validation and policy discussion.

The crisis of belonging among digital-native emerging adults is not an ephemeral cultural anxiety but a defining structural condition of the twenty-first century. When attention replaces empathy and competition supplants care, the emotional infrastructure of humanity collapses. Psychiatry’s future relevance depends on its ability to move from describing this collapse to rebuilding systems of relational trust that integrate ethical technology, policy reform, and community participation. Recognising relational safety as both a clinical construct and a civic right can guide a new generation of interventions that are technically feasible, socially acceptable, empirically validated, ethically compliant, and structurally sustainable. For this framework to achieve public-health relevance, conceptual rigour must be accompanied by forms of translation that render relational safety intelligible and actionable beyond academic settings. Youth-facing communication, co-produced dissemination strategies, and community-accessible summaries constitute essential extensions of this work, ensuring that relational safety and belonging are not only theoretically robust but also socially intelligible and practically actionable for the populations whose mental health trajectories they seek to shape. Belonging, when reimagined through this lens, becomes not a contingent outcome of stability but a foundational condition for collective mental health.
